# A Comparison Between Omicron and Earlier COVID-19 Variants' Disease Severity in the Milan Area, Italy

**DOI:** 10.3389/fepid.2022.891162

**Published:** 2022-06-28

**Authors:** David Consolazio, Rossella Murtas, Sara Tunesi, Anna Lamberti, Sabrina Senatore, Marino Faccini, Antonio Giampiero Russo

**Affiliations:** ^1^Epidemiology Unit, Agency for Health Protection of the Metropolitan City of Milan, Milan, Italy; ^2^Department of Sociology and Social Research, University of Milan-Bicocca, Milan, Italy; ^3^Preventive Medicine - Infectious Diseases Unit, Agency for Health Protection of the Metropolitan City of Milan, Milan, Italy

**Keywords:** COVID-19, SARS-CoV-2, Omicron, variants, vaccination, symptoms, hospitalization, negativization

## Abstract

**Background:**

In the context of the fourth wave of the COVID-19 pandemic in Italy, which occurred in correspondence with the outbreak of the Omicron variant, it became fundamental to assess differences in the risk of severe disease between the Omicron variant and the earlier SARS-CoV-2 variants that were still in circulation despite Omicron becoming prevalent.

**Methods:**

We collected data on 2,267 genotyped PCR-positive swab tests and assessed whether the presence of symptoms, risk of hospitalization, and recovery times were significantly different between Omicron and the earlier variants. Multivariable models adjusted for sex, age class, citizenship, comorbidities, and symptomatology allowed assessing the difference in outcomes between Omicron and the earlier variants according to vaccination status and timing of administration.

**Results:**

Compared to the earlier variants in the same period, Omicron was less symptomatic, resulted in fewer hospital admissions for those who were unvaccinated and for those who were already immunized after the booster dose, and was associated with quicker recovery, yet not in subjects with three vaccination doses.

**Conclusion:**

Despite being milder, Omicron's higher transmissibility and vaccine resistance should not lead to underrating its damage potential, especially with regard to hospital and health service saturation.

## Introduction

**Figure 1:**
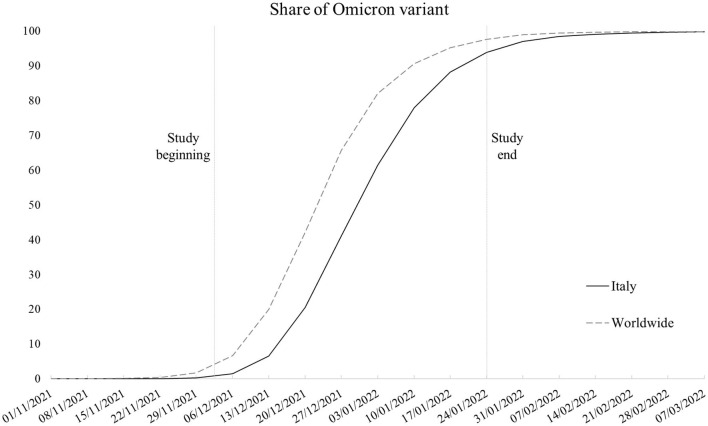
Smoothed weekly trend of share of SARS-CoV-2 sequences that are Omicron variant in Italy and worldwide (Italy excluded). Source: our elaboration of GISAID data (https://www.gisaid.org/).

The Omicron variant (lineage B.1.1.529) started to spread in Italy at the end of November 2021 within the context of an upswing in contagion due to the Delta variant (lineage B.1.167.2). Despite the prompt ban of flights from Southern African nations where the new variant of concern originated and introduction of more stringent limitations for unvaccinated people in public spaces, in a few weeks, Omicron caused the contagion curve and R_t_ to increase exponentially, contributing to the fourth local COVID-19 wave, with Omicron soon becoming the dominant variant (1). Figure 1 shows the weekly proportion of SARS-CoV-2 sequences (not cases) attributable to the Omicron variant since its appearance in the first week of November 2021 (2–4), comparing the Italian context with the rest of the world. As noticeable, worldwide, Omicron overtook the other variants in 9 weeks, with Italy following an analogous trend with a 1-week delay. In Italy, the novel strain accounted for about 1% of sequences in its first 2 weeks to 94% of the sequences at the end of the study period, coming to cover nearly all sequences in the following period. Despite the rising concern, according to the first evidence, the new variant appeared to be way more contagious but less symptomatic and severe than its predecessors (5–10), leading public opinion and some local authorities to underrate its damage potential. Accordingly, we examined data concerning positive-tested subjects whose nasopharyngeal swab tests were genotyped to identify the variant of belonging, with the aim to assess whether the presence of symptoms, risk of hospitalization, and recovery times substantially differs between Omicron and the previous dominant strains.

With regard to the Italian context, the monitoring of variants of concern followed international guidelines (11); however, locally, the absolute number of tests undergoing genomic sequencing may be limited (12). Therefore, the assessment of variants' severity is typically performed by comparing the overall COVID-19-related outcomes in different time spans assuming such outcomes to be related to the dominant variant in that specific period (13, 14). Following this approach, a country-level study identified a decreasing severity in relation to the Omicron wave compared to its predecessor (15). Despite its usefulness, this method allows for only indirect and potentially biased estimation of variants' severity, not being able to discern the share of one strain on the total number of cases in each specific period. This task has been effectively performed by means of wastewater surveillance (16), which does not allow matching information with patient data to assess the disease severity. Large-sample studies linking information on genome sequencing with individual outcomes have been conducted in South Africa (7), Norway (9), and the United Kingdom (17). To our knowledge, in Italy, the individual outcomes of infected patients with genotyped swab tests have been studied only in relation to an extremely small sample in the context of a local hospital outbreak, without comparing them with a sample of previous variants (18).

Therefore, this study constitutes the first attempt to comparatively investigate the disease severity of the novel variant within the Italian context.

## Methods

Samples of 2,267 PCR nasopharyngeal swab tests performed on subjects living in the territory of the Agency of Health Protection of the Metropolitan City (ATS) of Milan were genotyped to determine the specific SARS-CoV-2 variant between 2 December 2021 and 24 January 2022. We collected, through databases specifically developed since the beginning of the outbreak, information on the presence of typical COVID-19-related symptoms (19) (respiratory, fever, dyspnea, anosmia, ageusia, dysentery, muscle aches, asthenia, conjunctivitis, and headache), vaccination status, hospitalization, and recovery times (day difference between first positive test and first negative test). We obtained additional clinical and demographic information by record linkage to the Administrative Healthcare Databases of the ATS of Milan (sex, age, citizenship, and presence of comorbidities). The data were anonymized after the linkage. We restricted the analyses on subjects aged 18 years old or older to exclude those who were not eligible for vaccination in the whole enrollment period, which may have altered the results.

In order to evaluate the differences in symptom presentation between Omicron and the earlier variants, we first used a series of multivariable logistic regression models separately for each symptom as outcome. The models were adjusted for sex, age class (18–39, 40–49, 50–59, 60–69, and 70+), citizenship, number of comorbidities (0, 1+), and vaccination status. Second, we modeled the probability of hospitalization according to the variants in a multivariable logistic model adjusted for sex, age class, citizenship, number of comorbidities, vaccination status, and number of symptoms (from 0 to 9).

We further included in the model an interaction term to evaluate the differences in the odds of hospitalization between Omicron and the other variants according to vaccination status. Finally, we used an analogous model with time length of infection as outcome (linear regression) to assess the differences in recovery times between Omicron and the other variants, again according to vaccination status. Concerning vaccination, in all models we took into account not only the number of doses (from 0 to 3), but also the amount of time elapsed between the most recent dose and the infection. Immunity after vaccine is known to be activated 7–14 days after the dose (20) and to wane about 6 months after receipt (21, 22). In line with the literature (9, 10), we relied on the following categorization: unvaccinated; one dose <21 days before positive test; one dose more than 21 days before positive test; two doses 7–179 days before positive test; two doses more than 180 days before positive test; three doses <7 days before positive test; three doses more than 7 days before positive test. Statistical significance was set at 95% CIs.

## Results

**Figure 2:**
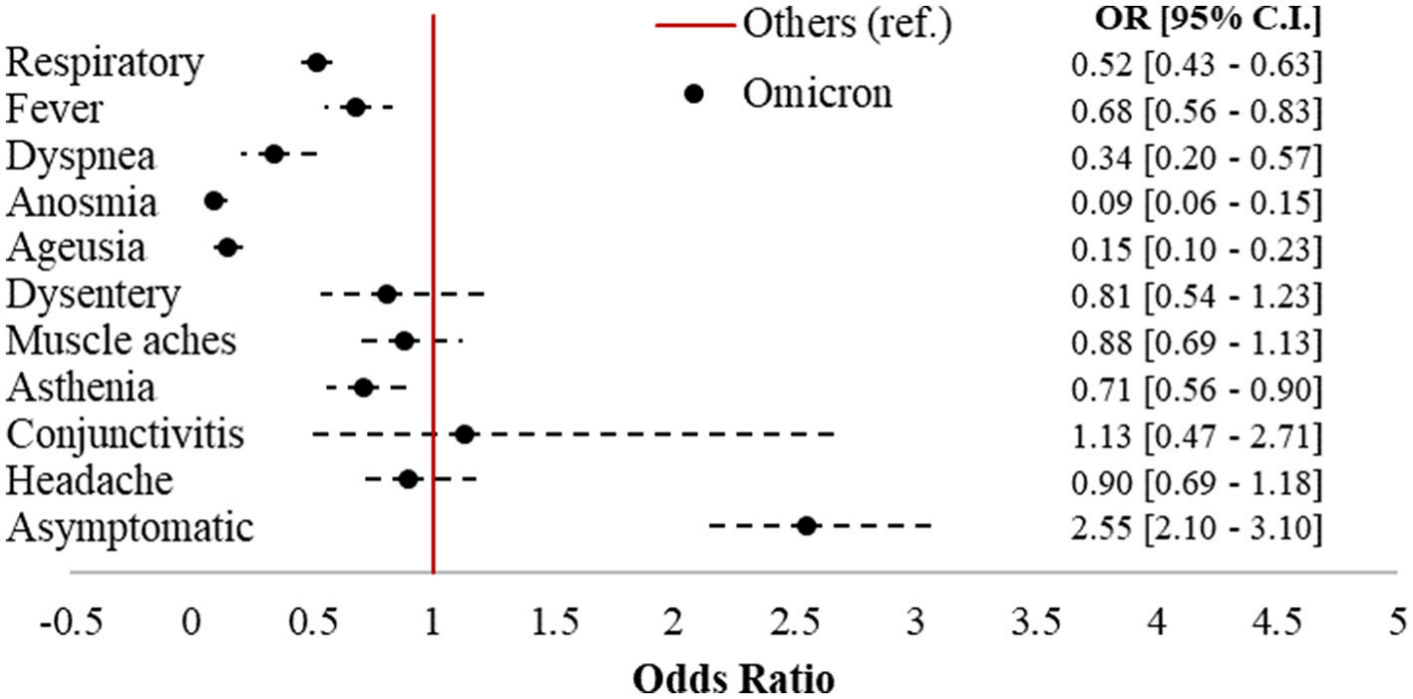
Forest plot of COVID-19-related symptoms in Omicron vs. other variants (ref.). Logistic regression models adjusted for sex, age class (18–39, 40–49, 50–59, 60–69, and 70+), citizenship (Italian, foreigner), number of comorbidities (0, 1+), and vaccination status (unvaccinated; one dose; two doses 7–179 dbpt*; 2 doses ≥ 180 dbpt*; 3 doses <7 dbpt*; 3 doses ≥ 7 dbpt*). ^*^dbpt, days before positive test.

Omicron accounted for 992 cases (43.8%) in the sample, followed by 802 Delta (35.4%), 362 Kappa (16%), and 111 (4.9%) cases belonging to other strains. Figure 2 displays the forest plot of the odds of each symptom in Omicron compared to the earlier variants. Omicron cases had 2.55 (95% CI = 2.1–3.1) times the odds of being asymptomatic compared to subjects affected by other strains, with significantly protective odds ratios (ORs) for six out of 10 of the symptoms investigated. The largest protective effects were detected for anosmia (OR = 0.09; 95% CI = 0.06–0.15) and ageusia (OR = 0.15; 95% CI = 0.1–0.23). Respiratory symptoms (OR = 0.52; 95% CI = 0.43–0.63), fever (OR = 0.68; 95% CI = 0.56–0.83), dyspnea (OR = 0.34; 95% CI = 0.2–0.57), and asthenia (OR = 0.71; 95% CI = 0.56–0.9) were also significantly less present in Omicron compared to the earlier variants.

**Figure 3:**
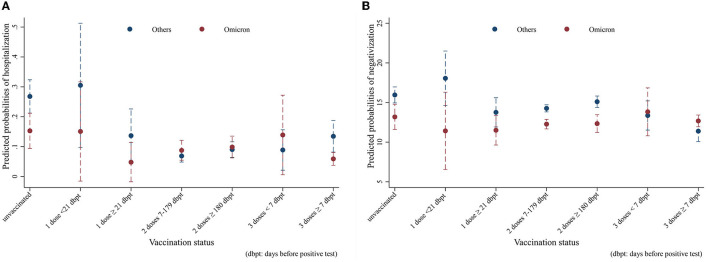
Average adjusted predictions (AAPs) of **(A)** hospitalization and **(B)** recovery time in Omicron vs. other variants by vaccination status (unvaccinated; one dose; two doses 7–179 dbpt*; 2 doses ≥ 180 dbpt*; 3 doses <7 dbpt*; 3 doses ≥ 7 dbpt*). Models were adjusted for sex, age class (18–39, 40–49, 50–59, 60–69, and 70+), citizenship (Italian, foreigner), number of comorbidities (0, 1+), and number of symptoms (0–9). *dbpt, days before positive test.

The results of the hospitalization and negativization models were reported in the form of average adjusted predictions (AAPs), expressing for each vaccination status category the predicted probability (computed *a posteriori*) of the outcome, net of the covariates included in the models. The non-overlapping 95% CI bars were interpreted as indicative of statistically significant differences. As Figure 3A shows, all other things being equal, Omicron unvaccinated cases (AAP = 0.15; 95% CI = 0.09–0.21) had a significant 12%-point lower probability of hospitalization compared to the analogs effect by the other variants (AAP = 0.27; 95% CI = 21–0.32). Among the vaccinated, only Omicron cases who had a booster dose more than 7 days before infection (AAP = 0.06; 95% CI = 0.04–0.08) resulted to be significantly at lower risk compared to their counterparts (AAP = 0.13; 95% CI = 0.08–0.19), with a 7%-point difference. With regard to negativization, Figure 3B shows a substantially quicker recovery for Omicron among the unvaccinated and those with two vaccine doses. In this case, the probabilities were predicted from a linear rather than logistic regression model; hence, the values coincide with the number of days to healing. Net of confounders, among the unvaccinated Omicron subjects (AAP = 13.2, 95% CI = 11.6–14.8) became negative on average almost 3 days earlier than those affected by other variants (AAP = 16; 95% CI = 15–17), while the difference was 2 days in those with two doses between 7 and 179 days before infection, and nearly 3 days in those with two doses more than 180 days after infection.

## Discussion

Compared to earlier variants, Omicron was less symptomatic and less subject to hospitalization in the unvaccinated and those who had received a booster shot at least seven days before the infection, as well as lasting for shorter time. Among the symptoms, anosmia and ageusia, the loss of smell and taste, respectively, which have been among the most present clinical manifestations of the disease since the beginning of the pandemic (23), were detected as the largest difference between the variants considered, although four other symptoms were also clearly less present in Omicron.

Despite resulting in a lower overall hospitalization rate (8.9% vs. 11.1%), patients with Omicron were significantly less likely to be hospitalized only among the unvaccinated and those already immunized with the third dose. This may highlight the effectiveness of the vaccination for the earlier variants, providing a level of protection able to overcome the differences in outcomes between forms of the disease characterized by different degrees of severity. Indeed, for subjects with earlier variants, each level of vaccination after the first dose was significantly associated with a reduction in the probability of hospitalization, whereas among Omicron cases, only those with a booster dose for at least 7 days were better protected. Our main findings regarding the risk of hospitalization are in line with those from a study conducted with a larger sample in Norway, which did not find a significant decrease in risk for persons with one or two vaccine doses in the comparison between Omicron and Delta cases (9), confirming the additional protection provided by the third dose against the novel variant (24, 25).

Regarding negativization, Omicron recovery times were significantly lower than earlier variants in the unvaccinated and in those with two doses. However, earlier variants showed a significant response to complete vaccination cycles (2 doses between 7 and 179 days before infection or three doses more than seven days before infection), as visible from the fact that recovery times in the fully vaccinated were always lower than in the unvaccinated or in those with one dose. This does not hold true for Omicron, for which there were no significant differences in recovery times according to vaccination status, highlighting a higher response to vaccination for earlier variants compared to Omicron.

To sum up, in line with pre-existing knowledge (5–10), Omicron emerged as a less severe variant. However, this study has some limitations that we need to highlight. First, as the data regarded a selected sample of positive-tested cases, we had no means to test the differences in disease transmission between Omicron and the earlier variants and the role of vaccination in mitigating their spread rather than just their outcomes. Second, despite the local sequencing being developed following the criteria established by the Italian National Institute of Health to obtain representative samples, the sampling strategy is constantly affected by the emergence of new strains. In correspondence with the emergence of the Omicron variant, swab tests of subjects returning from abroad (especially from African countries) were more likely to be tested because of the need to trace and contain its spread. This may have potentially led to oversampling of Omicron tests in the study period. Third, although it would have been of interest to test the models with outcomes more sensitive to disease severity, the low absolute number in terms of deaths (and the impossibility to determine the cause of death for infected patients in the short run) and intensive care admissions, which accounted for 45 (2%) and 6 (0.2%) cases in the overall sample, respectively, made it unfeasible to develop robust and meaningful models. Such low numbers are a consequence of the nature of the study design, with the data not referring to the whole population of the study area but rather to a sample. The analysis of mortality and intensive care admissions will constitute the next steps of the ongoing research when data on a larger sample will be available and once enough time has elapsed to identify COVID-19-specific mortality from healthcare databases. Fourth, the Omicron prevalence was not stable in the study period, ranging from representing between 1 and 41% of sequences in the first 4 weeks to between 61 and 94% of sequences in the last 4 weeks in Italy as a whole (refer to Figure 1). We are not aware if this may have altered the comparison with other variants in the same time interval.

As major strengths, we highlight that in line with other international studies we were able to link sequences with high-quality administrative healthcare data on individual outcomes, which is unprecedented in the Italian context. In addition, the study was conducted in a delimited period that was stable with regard to vaccination outcomes, seasonality trends, and healthcare options. Indeed, previous studies compared the impact of different strains in distinct waves characterized by marked heterogeneity in population vaccination rates and seasonality variations. In our case, the comparison took place more than a year after the beginning of the national vaccination campaign, also being less affected by fluctuations in climatic characteristics and variations in containment measures.

In conclusion, despite Omicron resulting in less severe forms of the disease, among the vaccinated, the reduction in hospitalization and recovery times bestowed by vaccination were more appreciable among those affected by the earlier variants than by Omicron. Hence, the combination of Omicron's increased transmissibility, and its higher vaccine resistance should not allow the underestimation of the disruptive potential of this new variant, which in the event of large waves of infection may contribute to the saturation of the healthcare system, with a considerable burden on hospitals and health services (6, 9). This should induce decision-makers to carefully evaluate the possibility of easing the containment measures (e.g., social distancing, mask mandate, limitations in access to public places, digital COVID-19 certificates, and the like), basing decisions not only on disease severity but also on its contagiousness.

## Data Availability Statement

The data analyzed in this study is subject to the following licenses/restrictions: the dataset generated and analyzed during the current study is not publicly available due to privacy concerns, as it originates from administrative healthcare databases, which are subject to privacy restrictions according to the local legislation. Requests to access these datasets should be directed to AR, agrusso@ats-milano-it, https://www.ats-milano.it.

## Ethics Statement

Ethical review and approval was not required for the study on human participants in accordance with the local legislation and institutional requirements. Written informed consent for participation was not required for this study in accordance with the national legislation and the institutional requirements.

## Author Contributions

DC: conceptualization, methodology, formal analysis, writing—original draft, writing—review and editing, and visualization. RM and ST: data curation, conceptualization, methodology, and writing—review and editing. AL, SS, and MF: conceptualization, writing—review and editing, and project administration. AR: data curation, resources, conceptualization, methodology, writing—review and editing, project administration, and supervision. All authors contributed to the article and approved the submitted version.

## Conflict of Interest

The authors declare that the research was conducted in the absence of any commercial or financial relationships that could be construed as a potential conflict of interest.

## Publisher's Note

All claims expressed in this article are solely those of the authors and do not necessarily represent those of their affiliated organizations, or those of the publisher, the editors and the reviewers. Any product that may be evaluated in this article, or claim that may be made by its manufacturer, is not guaranteed or endorsed by the publisher.
