# Supplementary material for: A Comparison Between Omicron and Earlier COVID-19 Variants' Disease Severity in the Milan Area, Italy
Source: Front Epidemiol. 2022 Jun 28;2:891162. doi: 10.3389/fepid.2022.891162 (PMC10910966; doi:10.3389/fepid.2022.891162)
Supplement: Supplementary file 1 [file Data_Sheet_1.docx]

## **Supplementary Material**

*Table S1: Descriptive statistics of the variables in the models by variant.*

|  | **Others** | | **Omicron** | | **Total** | |
| --- | --- | --- | --- | --- | --- | --- |
|  | **N** | **%** | **N** | **%** | **N** | **%** |
| **Sex** |  |  |  |  |  |  |
| *Female* | 718 | 56.3 | 515 | 51.9 | 1,233 | 54.4 |
| *Male* | 557 | 43.7 | 477 | 48.1 | 1,034 | 45.6 |
|  |  |  |  |  |  |  |
| **Age class** |  |  |  |  |  |  |
| *18-39* | 407 | 31.9 | 408 | 41.1 | 815 | 36.0 |
| *40-49* | 302 | 23.7 | 166 | 16.7 | 468 | 20.6 |
| *50-59* | 219 | 17.2 | 175 | 17.6 | 394 | 17.4 |
| *60-69* | 136 | 10.7 | 100 | 10.1 | 236 | 10.4 |
| *70+* | 211 | 16.6 | 143 | 14.4 | 354 | 15.6 |
|  |  |  |  |  |  |  |
| **Citizenship** |  |  |  |  |  |  |
| *Foreigner* | 88 | 6.9 | 72 | 7.3 | 160 | 7.1 |
| *Italian* | 1,187 | 93.1 | 920 | 92.7 | 2,107 | 92.9 |
|  |  |  |  |  |  |  |
| **Comorbidities** |  |  |  |  |  |  |
| *0* | 1,001 | 78.5 | 784 | 79.0 | 1,785 | 78.7 |
| *1+* | 274 | 21.5 | 208 | 21.0 | 482 | 21.3 |
|  |  |  |  |  |  |  |
| **Vaccination status** |  |  |  |  |  |  |
| *unvaccinated* | 168 | 13.2 | 87 | 8.8 | 255 | 11.3 |
| *1 dose < 21 dbpt** | 11 | 0.86 | 7 | 0.71 | 18 | 0.79 |
| *1 dose ≥ 21 dbpt** | 40 | 3.14 | 39 | 3.93 | 79 | 3.48 |
| *2 doses 7-179 dbpt** | 660 | 51.8 | 395 | 39.8 | 1,055 | 46.5 |
| *2 doses ≥ 180 dbpt** | 274 | 21.5 | 137 | 13.8 | 411 | 18.1 |
| *3 doses < 7 dbpt** | 39 | 3.1 | 13 | 1.3 | 52 | 2.3 |
| *3 doses ≥ 7 dbpt** | 83 | 6.5 | 314 | 31.7 | 397 | 17.5 |
|  |  |  |  |  |  |  |
| **Total** | 1,275 | 56.2 | 992 | 43.8 | 2,267 | - |

*^*^ dbpt: days before positive test*

*Table S2: Presence of symptoms by variant (N=2,267).*

|  | **Others** | | **Omicron** | |  | |
| --- | --- | --- | --- | --- | --- | --- |
|  | **N** | **%** | **N** | **%** | **N** | **%** |
| **Respiratory** | 755 | 59.2 | 411 | 41.4 | 1,166 | 51.4 |
| **Fever** | 434 | 34.0 | 222 | 22.4 | 656 | 28.9 |
| **Dyspnea** | 81 | 6.4 | 21 | 2.1 | 102 | 4.5 |
| **Anosmia** | 275 | 21.6 | 25 | 2.5 | 300 | 13.2 |
| **Ageusia** | 203 | 15.9 | 27 | 2.7 | 230 | 10.2 |
| **Dysentery** | 70 | 5.5 | 45 | 4.5 | 115 | 5.1 |
| **Muscle aches** | 234 | 18.4 | 142 | 14.3 | 376 | 16.6 |
| **Asthenia** | 286 | 22.4 | 153 | 15.4 | 439 | 19.4 |
| **Conjunctivitis** | 13 | 1.0 | 11 | 1.1 | 24 | 1.1 |
| **Headache** | 180 | 14.1 | 115 | 11.6 | 295 | 13.0 |
| **Asymptomatic** | 345 | 27.1 | 504 | 50.8 | 849 | 37.5 |

*Table S3: Hospitalization rates by variant and vaccination status (N=2,267).*

|  | **Others** | | **Omicron** | | **Total** | |
| --- | --- | --- | --- | --- | --- | --- |
|  | **N** | **%** | **N** | **%** | **N** | **%** |
| **Vaccination status** |  |  |  |  |  |  |
| *unvaccinated* | 43 | 25.6 | 17 | 19.5 | 60 | 23.5 |
| *1 dose < 21 dbpt** | 4 | 36.4 | 2 | 28.6 | 6 | 33.3 |
| *1 dose ≥ 21 dbpt** | 5 | 12.5 | 2 | 5.13 | 7 | 8.86 |
| *2 doses 7-179 dbpt** | 32 | 4.9 | 18 | 4.6 | 50 | 4.7 |
| *2 doses ≥ 180 dbpt** | 35 | 12.8 | 22 | 16.1 | 57 | 13.9 |
| *3 doses < 7 dbpt** | 5 | 12.8 | 3 | 23.1 | 8 | 15.4 |
| *3 doses ≥ 7 dbpt** | 17 | 20.5 | 24 | 7.6 | 41 | 10.3 |
| **Total** | 141 | 11.1 | 88 | 8.9 | 229 | 10.1 |

*^*^ dbpt: days before positive test*

*Table S4: Average time length of negativization by variant and vaccination status (N=1,859).
NB: N is lower than the total sample due to deceased subjects and subjects without a negative test at the enrolling date.*

|  | **Others** | | **Omicron** | | **Total** | |
| --- | --- | --- | --- | --- | --- | --- |
|  | **N** | **x̄** | **N** | **x̄** | **N** | **x̄** |
| **Vaccination status** |  |  |  |  |  |  |
| *unvaccinated* | 116 | 15.9 | 47 | 13.0 | 163 | 15.0 |
| *1 dose < 21 dbpt** | 10 | 18.4 | 5 | 11.2 | 15 | 16.0 |
| *1 dose ≥ 21 dbpt** | 35 | 13.7 | 34 | 11.0 | 69 | 12.4 |
| *2 doses 7-179 dbpt** | 607 | 14.2 | 336 | 11.7 | 943 | 13.3 |
| *2 doses ≥ 180 dbpt** | 237 | 15.8 | 95 | 12.7 | 332 | 14.9 |
| *3 doses < 7 dbpt** | 35 | 14.2 | 13 | 15.2 | 48 | 14.4 |
| *3 doses ≥ 7 dbpt** | 69 | 12.0 | 220 | 12.7 | 289 | 12.5 |
| **Total** | 1,109 | 14.6 | 750 | 12.1 | 1,859 | 13.6 |

*^*^ dbpt: days before positive test*
